# Are persistent organic pollutants important in the etiology of feline hyperthyroidism? A review

**DOI:** 10.1186/s13028-019-0478-9

**Published:** 2019-10-03

**Authors:** Bernt Jones, Jessica Norrgran Engdahl, Jana Weiss

**Affiliations:** 1 Department of Clinical Sciences, Swedish University of Agricultural Sciences, 750 07 Uppsala, Sweden; 2 Swedish Chemicals Agency, 172 13 Sundbyberg, Sweden; 3 Department of Environmental Science and Analytical Chemistry, Stockholm University, 114 18 Stockholm, Sweden

**Keywords:** Brominated flame retardants, Etiological factors, Feline hyperthyroidism, Organohalogen compounds, Persistent organic pollutants

## Abstract

Feline hyperthyroidism is a rather new disease, first reported from the North American east coast in 1979. The prevalence is increasing, especially in older cats, and hyperthyroidism is now reported worldwide as the most common feline endocrinopathy. Several studies have been performed trying to identify important etiological factors such as exposure to persistent organic pollutants, and especially brominated flame retardants, have been suggested to be of importance for the development of the disease. Recent studies have shown higher concentrations of these contaminants in serum of hyperthyroid cats in comparison to cats with normal thyroid status. However, other still unknown factors are most probably of importance for the development of this disease.

## Introduction

Before the end of the 1970s feline hyperthyroidism (FHT) was a rare disease generally caused by carcinomas of the thyroid gland resulting in an overproduction of thyroid hormones. However, in 1979 Peterson et al. [1] published a report on a new type of FHT with a still unknown etiology. Typically, FHT is more commonly seen in elderly cats (> 8 years), and rarely in younger cats (less than 5% of the cases).

Histologically the thyroid gland may demonstrate follicular adenoma or a multinodular hyperplasia in cases with FHT. Adenoma and hyperplasia may occur simultaneously within the same thyroid gland [2]. Since the first report from the North American east coast, this new disease has now been reported from all continents and it is showing an increasing prevalence [3–6]. In North America, FHT has increased from 0.3% in 1979 to 4.5% in 1985, whilst another study reported an increase from 0.1% in 1978–1982 to 2% in the period 1993–1997 [5, 7]. In an English study (n = 2276 cases) the overall prevalence of FHT was 2.4%, but in older cats (> 10 years) the prevalence was 8.7% [8].

At the same time, the severity of clinical signs, typically weight loss, polyphagia, polydipsia, hyper-activity, aggression, diarrhea, vomiting and tachycardia [9] has decreased, because of an increased awareness of FHT in the veterinary profession [10]. The ever-higher age of the cat population due to an increase in social status and better care in Europe, North America, Australia and Japan may account for some of the increasing number of FHT cases. However, this reasoning does not solely explain the increasing prevalence of FHT seen worldwide [7].

The physiological effects of the thyroid hormones T_4_ (thyroxine, 3,3′,5,5′-tetraiodo-l-thyrone) and T_3_ (triiodothyronine, 3,3′,5-tetraiodo-l-thyronine) on the mammalian metabolism are similar in all mammalian species studied. However, cats differ from other vertebrate species in respect of the plasma proteins transporting thyroid hormones. Cats appear to lack the protein thyroxin binding globulin (TBG), which is an important transport protein in other mammalians, therefore transthyretin (TTR) becomes the main thyroid transporting plasma protein [11].

In 2009, Dye et al. [12] introduced the hypothesis that anthropogenic chemicals could explain the increased numbers of reported FHT cases since the occurrence of FHT started in parallel with the introduction of flame retardants in household products, e.g. the polybrominated diphenyl ethers (PBDEs). The question was raised if these compounds have an etiological role and consequently, several studies started to analyze for anthropogenic chemicals in cat serum to search for a cause-effect relationship [12–25].

## Search strategy

This critical review is summarizing the existing studies on persistent organic pollutants analyzed in cat samples, and is putting the gathered information in perspective to FHT, metabolism and other etiological studies. The search for relevant literature in PubMed (http://www.ncbi.nlm.nih.gov/PubMed) was using the terms “cat” and “feline” in combination with terms such as; “persistent organic pollutant, organohalogen compound, pesticide (HCH, PCP, DDT etc.), PCB, flame retardant (incl. subgroups such as brominated FR, organophosphorus FR, PDBE, HBCD, TBBPA, TBP etc.), hydroxylated metabolites, phenolic compound, poly- and perflouroalkyl substances (PFAS, incl. PFOA and PFOS), etc.” For the general discussion regarding feline hyperthyroidism, thyroid functionality and metabolism no general search strategy was applied. The authors’ gathered expertise in the field was used to find literature where knowledge gaps demanded it.

## Feline metabolism of xenobiotics

Cats are hyper carnivores and have lower activity of certain cytochrome P450-enzymes involved in both phase I and II reactions, hence limiting their ability to metabolize certain xenobiotics [26]. In particular, the conjugation enzymes glucuronyl transferase and sulphotransferases in phase II have lower activities in cats compared to e.g. dogs and humans [27]. Sulfation of T_4_ appear to be insignificant in cats according to a study on thyroid hormone transport across the feline gut wall, which is an important route of elimination of T_4_ [28]. This will affect the metabolism and elimination of most xenobiotic compounds in the cat, including natural plant substances and toxins, substances easily metabolized by other animals. Glucuronidation accounts for detoxification of a number of phenolic substances in other species but clearance of these substances are slower in cats. A well-known example of this is the toxicity in cats of acetaminophen which is not toxic to dogs or humans in therapeutic doses. Glucuronides are also the main bile excretion form of T_4_ in cats [29], but if this has any etiological importance in FHT is not known. In euthyroid cats with normal thyroid function, excretion of glucoronidated T_4_ is obviously sufficient but if this mechanism is overloaded in FHT and if this further aggravate the condition is to our knowledge not known.

## Epidemiology

Several epidemiological studies have been performed trying to identify important etiological factors explaining FHT. Even though several hypotheses have been tested, no single factor has yet been proved in these studies explaining the outbreak of this new disease.

In an early North American study, it was found that cats with FHT more often used litter boxes, had been treated for ectoparasites and were fed canned food [4]. The more frequent use of litter boxes was also observed by Wakeling et al. [6] as a risk factor for FHT, although this is probably more likely an indicator of a cat with an indoor life, as stated by the authors. In the study by Kass et al. [4] it was found that Siamese and Himalayan breeds were less prone to be affected by FHT, indicating that genetics are important for the development of FHT. Martin et al. [30] and Stephens et al. [8] also found that the Siamese breed had less risk of developing FHT. In the latter and more extensive study also Burmese, Persian and purebred cats were found to have an overall lower prevalence of FHT. In a New Zealand study, purebred cats also showed a lower risk than domestic short- and long-haired cats [31]. Further, these authors also suggested that females were at higher risk of developing FHT, which is not shown in other studies where no differences have been seen between genders (e.g. [7, 8]). In humans, females are at much higher risk developing toxic adenomatous goiter, which is the human equivalent of FHT [9].

Canned food was identified as a risk factor in several studies [6, 7, 30, 31]. The studies by Martin et al. [30] and Wakeling et al. [6] identified especially the fish flavored canned food to be associated to a higher incidence of FHT. On the other hand, De Wet et al. [3] found no correlation to the type of food eaten by the cats and FHT. However, more than 90% of the ingredients in cat food from the same producer is identical for all the different flavors [32].

Commonly, affected cats live a predominantly indoor life and have a high intake of canned food. A possible risk factor in the canned food is bisphenol A (BPA) a known endocrine disrupting compound (EDC) [33]. The content of BPA was analyzed in 15 canned Japanese cat food samples and found to be 60- to 70-fold below the limits established for human food by EU and Japanese authorities [34]. However, BPA has not been shown to bind to the transport protein TTR, which makes BPA less probable as an important etiological factor for FHT [47]. In addition, other possibly goitrogenic factors associated with the food have been identified in epidemiological studies performed around the world. Different vegetable substances like isoflavones and phthalates coming from soy and corn are identified as possible goitrogenic compounds in cat food [4, 26, 34, 35]. Isoflavones were determined in New Zealand cat food by Bell et al. [26] and found in concentrations known to have endocrine disrupting effects in other species. Several of these vegetable substances are eliminated via the glucuronidation pathway, a process known to be slow in cats due to their low activity of the enzyme glucuronyl transferase [27].

In commercial cat foods highly variable iodine content was found, an element essential for the synthesis of thyroid hormones [7]. In some cases the iodine content was clearly deviating from the recommendations given by the National Research Council (NRC) and Association of American Food Control Officials (AAFCO) for cat food.

In a review from South Africa it was suggested that there is a geographical difference in the incidence of FHT based on the different figures found in the open international literature [36]. However, Stephens et al. [8] found no significant differences between different regions of England. It is possible that the different figures from different countries presented in the literature are due to differences in data collection and classification of cases by the reporting clinicians.

The disparity of results obtained in epidemiological studies are difficult to evaluate due to e.g. different methods used to define study populations and other factors associated with the design of the studies. Furthermore, the recollection of the owner’s memory of living conditions, feeding habits of their cat several years ago is of crucial importance. However, a prudent evaluation of the different etiological factors found in published studies is that FHT is a multifactorial disease.

## Endocrine disrupting chemicals in cats

After the report by Dye et al. [12], domestic cats have been used as sentinels for human exposure to indoor pollutants like persistent organic pollutants (POPs), and especially the PBDEs and polychlorinated biphenyls (PCBs). Hydroxylated metabolites of these halogenated compounds have structural similarities of the natural thyroid hormones T_4_ and T_3_ [33, 37]. Analyses of cat blood serum from different parts of the world have shown high levels of PBDEs, higher than found in humans from the same area [12, 15, 18, 19, 22, 24]. The usage of PBDEs were introduced to the market just prior to the first cases of FHT were recorded [38]. These pollutants are emitted from electrical and electronic equipment (computers, TV-sets etc.), furniture and textiles. The reason for cats being especially exposed to these environmental pollutants is their natural behavior to regularly groom their fur coat, thereby removing and ingesting dust particles [14, 19, 20, 39]. Dust is a matrix collecting volatile and semi-volatile chemicals released from the indoor products, flooring, textiles, furniture and any human activity (skin, hair) [40]. Toddlers will be exposed to these compounds to a higher degree than adults in the same household due to the dust ingestion associated to their hand-to-mouth behavior. Studies in California [14] and Sweden [41] have shown that cats have approximately 50 times higher concentrations of PBDE in their blood than human adults from the same geographical area.

The structural similarities between OH-metabolites of PBDEs and PCBs and thyroid hormones have initiated studies trying to find correlations between the concentrations of these compounds in cat blood and FHT [14, 16, 17, 20, 42]. Two studies have been able to statistically show a significant correlation between cats with FHT and the blood concentration of some PBDEs and PCBs. The congeners associated with FHT differed between the studies. Norrgran et al. [20] found association to BDE-99, BDE-153, BDE-183, and CB153, whereas Walter et al. [25] found associations to BDE17, BDE100, BDE47, BDE49 and CB131, CB153, CB174, CB180 and CB196. The latter study analyzed serum from cats living in the US and reported total median levels eight times higher than the first study analyzing serum from cats living in Sweden. The pattern of PBDE congeners found in cats and humans differ, suggesting different exposure sources of the pollutant [14, 19, 43] and/or different metabolic capacity. The pattern of PBDEs found in cat serum is similar to the pattern found in dust suggesting dust to be the exposure source. In the studies by Chow et al. [19, 44] and Guo et al. [14] no correlation was found between house dust levels, serum levels and the thyroid status of matched cats, possibly because the serum concentrations were not age adjusted for.

In a later study by Norrgran Engdahl et al. [39] paired samples of cat serum, feed and house dust were used, a significant correlation between serum levels and dust concentrations were found. This was the first time that dust could be confirmed as being a relevant exposure pathway of these compounds, although it has been hypothesized earlier as the major contamination source for POP in cats [14, 44].

In addition, the Swedish study analysed 28 different cat food brands (dry and wet cat food), and found a significant correlation between cat serum and cat food concentrations for the flame retardant decabromobiphenyl (BB209) and two phenolic compounds 2,4,6-tribromophenol (2,4,6-TBP) and 6-hydroxy-2,2′,4,4′tetrabromo-diphenylether (6-OH-BDE47), suggesting cat food to be an important exposure pathway for these compounds [39]. Both 2,4,6-TBP and 6-OH-BDE47 are natural compounds formed by marine plants and may enter the food chain via sea food [45, 46]. Further, it was shown that cat liver microsomes were unable to form the 2,4,6-TBP metabolite from the parent PBDEs by incubation, indicating that cat food may be the source of the 2,4,6-TBP found in serum [24]. These phenolic compounds have a high binding affinity to the transport protein TTR and can competitively replace the natural ligand to be transported to its target tissue [47]. Also, these compounds could be found at higher concentration and more frequently in wet food compared to dry food. This is in accordance with previous epidemiological observations that cats eating wet/canned food had a higher prevalence for FHT [6, 7, 30, 31]. In fact, serum levels of 6-OH-BDE47 were 2–50 times higher in Japanese stray cats compared to cats living in UK and Sweden, which could support this association as their food is likely more influenced by fish from the ocean [17, 18, 20, 39].

Other compounds with potency to bind to TTR are the per- and polyfluoroalkyl substances (PFASs), which are ubiquitously found in our indoor environment, coming from products with functions such as water and grease repellents (food packaging, kitchen utensils and outdoor clothing) or low surface tension (sprays in cleaning products, floor polishing etc.) [47]. Cats from the US [21, 48] had 2–4 times higher serum levels than cats from Sweden [49]. The US study could show that serum from hyperthyroid cats showed higher PFAS level (9.50 ng/mL) compared to nonhyperthyroid cats (7.24 ng/mL), due to the levels of perfluorooctanoic acid (PFOA, P < 0.05). The Swedish study could demonstrate an association between serum levels of PFOA with the dust concentrations in their homes, confirming dust to be a relevant exposure pathway [49].

Recently, the non-halogenated organophosphate ester flame retardants (OPFR) was analyzed in cat serum [23]. It has been suggested that OPFR can enhance the T_4_ binding to human TTR, a mechanism not totally understood yet [50]. If this is also an effect in cats is not known to our knowledge. Many more chemicals, with TH disrupting potency and analyzed in dust have not yet been searched for in cat serum, e.g. phthalates, BPA, musk compounds, and parabens, commonly found in personal care products [39]. Considering the growing evidence of the endocrine effects of these compounds, and the increasing number of monitoring data in dust and serum (both for cats and humans) it is reasonable to attribute chemical exposure as contributing to the observed increased incidence of endocrine disorders in cats and humans, such as FHT.

## Conclusions

The correlation between pet cats spending the majority of time indoor, hence having a considerable dust intake, and the increase in reported incidence of FTH could possibly be associated to the vast number of chemicals analyzed in house dust, several of them showing thyroid hormone disrupting potency. The correlation to cats eating wet/canned food and FHT may be associated to the naturally occurring 6-OH-BDE-47 and 2,4,6-TBP derived from the marine environment, or the presence of other possible phenolic compounds not yet identified in the wet cat food. Although there are indications that anthropogenic chemicals, such as PBDEs and other POPs, could have etiological importance for the development of FHT a plausible time course for development of the disease, and more detailed biochemical mechanisms are needed to fully elucidate the mode of action behind this rather new disease. A thorough understanding of their role in the etiopathogenesis of FHT would need longitudinal studies. To protect our animals, and our children it is of outermost importance to identify and regulate the mixture of EDCs in our indoor environment.

## Data Availability

Not applicable.

## Abbreviations

AAFCO: Association of American food control officials
BDE: brominated diphenyl ethers
BPA: bisphenol A
EDC: endocrine disrupting compound
FHT: feline hyperthyroidism
NRC: National Research Council
OPFR: organophosphate ester flame retardants
PBDE: polybrominated diphenyl ethers
PCB: polychlorinated biphenyl
POP: persistent organic pollutants
T_3_: triiodothyronine
T_4_: thyroxine
TTR: transthyretin
